# Extracellular Signal-Regulated Kinase Mediates Ebastine-Induced Human Follicle Dermal Papilla Cell Proliferation

**DOI:** 10.1155/2019/6360503

**Published:** 2019-02-11

**Authors:** Fu-Ming Tsai, Chao-Hsu Li, Lu-Kai Wang, Mao-Liang Chen, Ming-Cheng Lee, Yi-Ying Lin, Chun-Hua Wang

**Affiliations:** ^1^Department of Research, Taipei Tzuchi Hospital, The Buddhist Tzuchi Medical Foundation, New Taipei City 231, Taiwan; ^2^Division of General Surgery, Department of Surgery, Taipei Tzuchi Hospital, The Buddhist Tzuchi Medical Foundation, New Taipei City 231, Taiwan; ^3^Graduate Institute of Clinical Medicine, College of Medicine, National Taiwan University, Taipei 100, Taiwan; ^4^Radiation Biology Core Laboratory, Institute for Radiological Research, Chang Gung University/Chang Gung Memorial Hospital, Linkou, Taoyuan 333, Taiwan; ^5^Department of Dermatology, Taipei Tzuchi Hospital, The Buddhist Tzuchi Medical Foundation, New Taipei City 231, Taiwan; ^6^School of Medicine, Tzu Chi University, Hualien 970, Taiwan

## Abstract

Ebastine is a second-generation histamine H1 receptor antagonist that is used to attenuate allergic inflammation. Ebastine has also shown to affect hair loss; however, the immunoregulatory effect of ebastine cannot completely exclude the possibility of spontaneous hair regrowth in ebastine-treated mice. In this study, we examined the effects of ebastine on the growth of human follicle dermal papilla cells (HFDPC) using a WST-1 cell proliferation assay and a bromodeoxyuridine incorporation assay. Ebastine was shown to significantly increase the proliferation of HFDPC. The expression levels of cell-cycle regulatory proteins and an antiapoptotic protein were increased in ebastine-treated HFDPC. Furthermore, elevated expression levels of phospho-AKT and phospho-p44/42 extracellular signal-regulated kinase (ERK) were observed in ebastine-treated HFDPC. Ebastine-mediated HFDPC growth was completely reversed by blocking ERK kinase. The results from our present study suggest that the regulation of HFDPC proliferation by ebastine might be directly involved in hair regrowth through the ERK signaling pathway.

## 1. Introduction

Histamine exerts its biological effects by binding and activating four G protein-coupled histamine receptors, named H1 through H4 [1]. The histamine H1 receptor is expressed in smooth muscles, vascular endothelial cells, the heart, the central nervous system, and mesenchymal stem cells [2]. Ebastine is among the most widely used antihistamines for attenuating the symptoms of seasonal and perennial allergic rhinitis. Ebastine is a second-generation histamine H1 receptor antagonist that inhibits allergen-induced afflictions, including bronchospasms, rhinitis, and chronic idiopathic urticaria [3–6]. Second-generation H1 antihistamines, such as ebastine and fexofenadine, are much more selective for peripheral H1 receptors, whereas nonselective first-generation H1 antihistaminergic drugs bind to acetylcholine receptors, *α*-adrenergic receptors, and 5-HT receptors [7, 8].

The major application of antihistamines is the control of symptoms associated with allergic reactions. Most pharmacological studies of antihistamines have focused on cellular aspects of allergic responses and immune regulation [9–12]. Nonetheless, some clinical observations have suggested favorable effects of antihistamines on alopecia areata [13–15]. Nori et al. showed that ebastine suppresses the production of Th2 and proinflammatory cytokines and inhibits T cell migration [16]. Ohyama et al. provided a rational basis for treating alopecia areata with antihistamines. T cell infiltration can hardly be detected in alopecia areata patches of ebastine-treated mice. This observation suggested that ebastine might play some roles in the suppression of the T cell-mediated immune response in alopecia areata lesions [14]. However, the role of ebastine in hair cell regrowth in alopecia areata lesions has not been addressed.

Dermal papilla cells are specialized fibroblasts of mesenchymal origin expressed in the base of hair follicles that are crucial for the induction of hair follicle growth [17]. Although ebastine exhibits activities that can regulate hair regrowth, the exact mechanism has not been determined. In this study, we investigated the effects of ebastine on the growth of human follicle dermal papilla cells (HFDPC) and characterized the underlying mechanisms, including the role of AKT and extracellular signal-regulated kinase (ERK) through the epidermal growth factor receptor pathway.

## 2. Materials and methods

### 2.1. Cells

HFDPC were purchased from PromoCell (Heidelberg, Germany). The cells were maintained in follicle dermal papilla cell basal medium supplemented with 4% fetal calf serum, 0.4% bovine pituitary extract, 1 ng/mL basic fibroblast growth factor, and 5 *μ*g/mL recombinant human insulin (PromoCell) at 37°C in 5% CO_2_.

### 2.2. Alkaline Phosphatase Activity Assay

Alkaline phosphatase activity was assessed by a biochemical colorimetric assay using an alkaline phosphatase assay kit (GeneTex Inc., Irvine, CA, USA). Briefly, the 4th through the 10th HFDPC population doublings were homogenized in the assay buffer through a syringe fitted with a 21-gauge needle. After centrifugation at 14,000 × g for 5 min, protein in the lysate was quantified using a Bio-Rad protein assay kit (Bio-Rad Laboratories, Hercules, CA, USA). Alkaline phosphatase activity was determined according to the manufacturer's instructions, and the quantified results were normalized to the protein concentration.

### 2.3. RNA Isolation and Quantitative Real-Time Reverse Transcription Polymerase Chain Reaction

Total RNA was extracted using TRI_ZOL_ reagent (Invitrogen, Carlsbad, CA, USA) according to the manufacturer's instructions. cDNA was prepared by incubating a mixture containing 3 *μ*g total RNA, 1 U Moloney murine leukemia virus (MuLV) reverse transcriptase (Invitrogen), 0.5 *μ*g oligo-dT_12-18_, 4 *μ*l 5× RT buffer, 0.5 mM dNTP, and 1 U RNaseOUT recombinant RNase inhibitor in a total volume of 20 *μ*l at 37°C for 1 h. Quantitative real-time PCR (Q-PCR) was performed in triplicate in a reaction mixture that was 25 *μ*l in total volume containing 12.5 *μ*l SYBR Green Master Mix (Applied-Biosystems, Foster City, CA, USA), 50 ng cDNA, and gene-specific forward and reverse primers at a final concentration of 1 *μ*M in a thermal cycler (7900HT Fast Real-Time PCR System, ABI). The PCR cycling program consisted of an initial incubation at 95°C for 3 min, 45 cycles of denaturation at 95°C for 15 s, and annealing and extension at 60°C for 1 min. The PCR primers used for amplification were as follows: *β*-actin (sense, 5'-TCCCTGGAGAAGAGCTACG-3' and antisense, 5'-GTAGTTTCGTGGATGCCACA-3'), histamine receptor H1 (sense, 5'- GTCTAACACAGGCCTGGATT-3' and antisense, 5'- GGATGAAGGCTGCCATGATA -3'), H2 (sense, 5'- ATTAGCTCCTGGAAGGCAGC -3' and antisense, 5'- CTGGAGCTTCAGGGGTTTCT -3'), H3 (sense, 5'- TCGTGCTCATCAGCTACGAC -3' and antisense, 5'- AAGCCGTGATGAGGAAGTAC -3'), H4 (sense, 5'- GGCTCACTACTGACTATCTG -3'), and antisense, 5'- CCTTCATCCTTCCAAGACTC -3'). The relative expression levels of the target cDNAs were quantified and normalized to the expression level of *β*-actin.

### 2.4. Cell Viability Assay

The 4th through the 10th HFDPC population doublings were seeded at 3 × 10^4^ cells/well in triplicate in 24-well plates. HFDPC were incubated with serum-free basal medium for 18 h and then cultured in medium containing 0.5% fetal calf serum with various concentrations of ebastine in the presence or absence of 5 *μ*M AKT1/2 kinase inhibitor (Santa Cruz Biotechnology, Santa Cruz, CA, USA) or 50 *μ*M PD98059 (Santa Cruz Biotechnology) for an additional 24 h. Cells were cultured in serum-free basal medium or incubated with complete growth medium as negative and positive controls, respectively. A WST-1 assay was used to analyze cell viability. Briefly, 100 *μ*L of WST-1 reagent (Roche Diagnostics, Mannheim, Germany) was added to each well, and the HFDPC were further incubated at 37°C for 4 h. Cells treated with WST-1 were then transferred to a 96-well plate, and the absorbance of each well was measured at wavelengths of 450 and 650 nm in a multifunctional microplate reader (Infinite F200, Tecan, Durham, NC, USA). The percentage of cell viability relative to the vehicle-treated cells was calculated as follows: [(A450–A650) of the drug-treated cells/(A450–A650) of the vehicle-treated cells] × 100%.

### 2.5. Cell Proliferation Assay and Cell Cycle Analysis

Cell proliferation was assessed with a bromodeoxyuridine (BrdU) incorporation kit from Roche Diagnostics. Briefly, the 4th through the 10th HFDPC population doublings were seeded at 1 × 10^4^ cells/well in triplicate in 24-well plates. HFDPC were incubated with serum-free basal medium for 18 h and then cultured in medium containing 0.5% fetal calf serum with various concentrations of ebastine in the presence or absence of 5 *μ*M AKT1/2 kinase inhibitor or 50 *μ*M PD98059 for an additional 48 h. Cells were cultured in serum-free basal medium or incubated with complete growth medium as negative and positive controls, respectively. Next, 10 *μ*M BrdU labeling solution was added to each well, and the mixture was incubated for 12 h. After removing the BrdU labeling solution, the cells were fixed and denatured with FixDenat solution for 30 min at 25°C. After the samples were fixed, they were incubated for 90 min at 25°C with a peroxidase-labeled anti-BrdU antibody and then incubated with tetramethyl-benzidine substrate. The color reaction was developed, and the optical densities of the samples were measured in a multifunctional microplate reader (Infinite F200), where the absorbance of each well was measured at 370 nm and 492 nm. For cell cycle analysis, HFDPC were incubated with serum-free basal medium for 18 h and then cultured in medium containing 0.5% fetal calf serum with various concentrations of ebastine for an additional 48 h. The cells were harvested and fixed in ice-cold 80% ethanol at -20°C overnight. The cells were stained with propidium iodide (Sigma-Aldrich, St. Louis, MO, USA) and then analyzed by flow cytometry (Cytomics FC 500; Becton Dickinson, Franklin Lakes, NJ) with an excitation wavelength of 488 nm and an emission wavelength of 620 nm. The percentage of cell cycle phase distribution was analyzed by Multicycle for Windows (Becton Dickinson).

### 2.6. Western Blotting

The cells were lysed in RIPA buffer (50 mM Tris [pH 8.0], 150 mM NaCl, 1% NP-40, 0.5% sodium deoxycholate, and 0.1% SDS) containing a protease inhibitor cocktail (Roche Diagnostics, Germany) and phosphatase inhibitors. Proteins (20-60 *μ*g) were separated on 12% polyacrylamide gels and transferred to polyvinylidene fluoride membranes. After the membranes were blocked, they were incubated with anti-histamine H1 receptor (GeneTex Inc), antihistamine H2 receptor (GeneTex Inc), anti-Cyclin D1 (Cell Signaling Technology, Beverly, MA, USA), anti-Cyclin E1 (Cell Signaling Technology), anti-Cyclin A (Cell Signaling Technology), anti-Cdk2 (Cell Signaling Technology), anti-Cdk4 (Cell Signaling Technology), anti-Cdc2(Cell Signaling Technology), anti-Bcl-2 (Santa Cruz Biotechnology), anti-Bax (Santa Cruz Biotechnology), anti-p-AKT (Cell Signaling Technology), anti-AKT (Cell Signaling Technology), anti-p-p44/p42 ERK (Cell Signaling Technology), anti-p44/p42 ERK (Cell Signaling Technology), and anti-Actin (Sigma-Aldrich) antibodies at 4°C for 12 h. The membranes were then incubated with corresponding horseradish peroxidase-conjugated secondary antibodies (Calbiochem, Darmstadt, Germany) at room temperature for 1 h. Specific protein bands were developed using Amersham ECL (Amersham, Bucks, UK). Relative protein expression was quantified and normalized to the expression levels of the Actin protein.

### 2.7. Statistical Analysis

Data are presented as the means ± SD of at least three replicates. Statistical analysis was performed using one-way ANOVA with Dunnett's post hoc test. A* p*-value < 0.05 was considered statistically significant.

## 3. Results

### 3.1. Ebastine Increased the Proliferation of HFDPC

**Figure 1:**
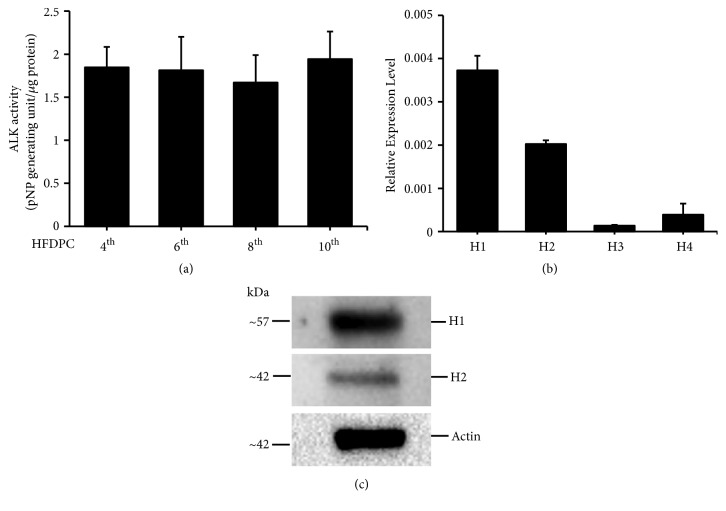
HFDPC expressed mRNA and proteins for several histamine receptor subtypes. HFDPC lysates were prepared and analyzed for alkaline phosphatase activity. Representative results from triplicate samples are presented as the mean ± SD (a). RNA from HFDPC was collected, and the expression levels of the histamine receptor subtypes were analyzed using real-time RT-PCR. After normalizing to *β*-actin, the relative expression levels are shown (b). HFDPC lysates were prepared, and the expression levels of histamine H1 and H2 receptors were determined by western blot analysis using H1 and H2 antibodies, respectively (c).

HFDPC were incubated and maintained in HFDPC basal medium supplemented with specific growth factors. We did not observe morphological changes (data not shown) or reduced alkaline phosphatase activity (Figure 1(a)) in the cultured cells. Ebastine is a receptor antagonist that mainly inhibits the activation of histamine receptors. To analyze the gene expression of histamine receptor subtypes in HFDPC, mRNA was collected from HFDPC and analyzed by real-time RT-PCR. We observed that the histamine H1 and H2 receptors were highly expressed in HFDPC, whereas the H3 and H4 subtypes were observed at low expression levels (Figure 1(b)). Furthermore, the protein expression levels of the histamine H1 and H2 receptors in HFDPC were determined (Figure 1(c)).

**Figure 2:**
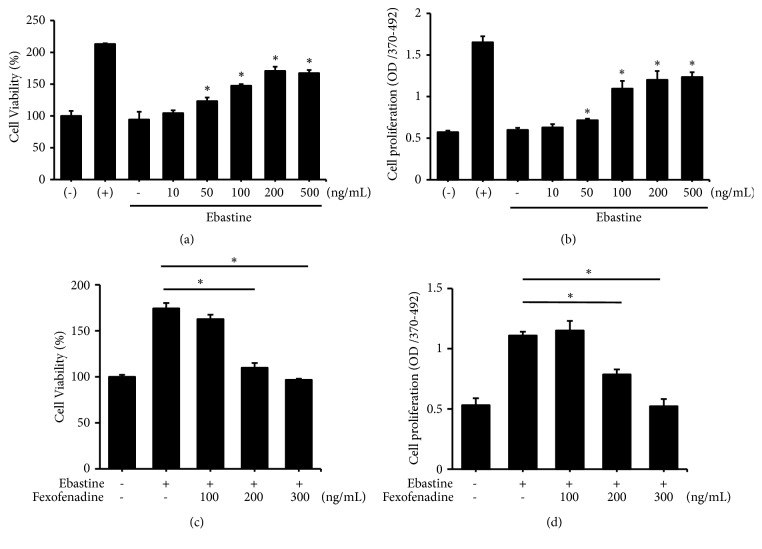
Ebastine-induced HFDPC growth. HFDPC were plated in triplicate in 24-well plates for 24 h. The cells were serum starved for 18 h and then treated with the indicated concentrations of ebastine ((a) and (b)) or 200 ng/mL ebastine along with indicated concentrations of fexofenadine for 24-48 h ((c) and (d)). Cell viability ((a) and (c)) and cell proliferation ((b) and (d)) were analyzed by WST-1 and bromodeoxyuridine (BrdU) assays, respectively. Representative results from three independent experiments are shown, and the data are presented as the mean ± SD after being normalized to the control group. *∗* indicates p < 0.05.

To assess the effects of ebastine on HFDPC, we first examined the viability of HFDPC treated with various doses of ebastine. Ebastine treatment of HFDPC did not appear to influence cell death, as indicated by lactate dehydrogenase activity (data not shown). However, a 16.9–75.6% increase in the number of viable cells was observed when HFDPC were treated with 50–500 ng/mL ebastine, with the maximum increase observed in cells treated with 200 ng/mL ebastine (Figure 2(a)). A BrdU incorporation assay was then used to determine whether the increased number of viable cells after ebastine treatment of HFDPC was related to cell proliferation. The proliferative activity in cells treated with 50–500 ng/mL ebastine was significantly enhanced, ranging from 16.9 to 101.6% of that of the control cells (Figure 2(b)). These results indicated that the concentration of ebastine in patient blood (the plasma levels of ebastine have been reported to be 68-151 ng/mL when ebastine was administered daily at a dose of 10 mg) may directly increase HFDPC proliferation [18]. The effect of fexofenadine (an antagonist to histamine H1 receptor) on the increased number of viable cells of HFDPC by ebastine was analyzed. Increased number of viable cells in HFDPC treated with ebastine was blocked by fexofenadine in a dose-dependent manner (Figure 2(c)). Also, the effect of ebastine on cell proliferation was suppressed when HFDPC-treated with higher doses of fexofenadine (Figure 2(d)). These observations indicated that the activity for ebastine induced-HFPDC proliferation is histamine H1 receptor dependent.

### 3.2. Ebastine Increased the Expression Levels of Cyclins and Cyclin-Dependent Kinases in HFDPC

**Figure 3:**
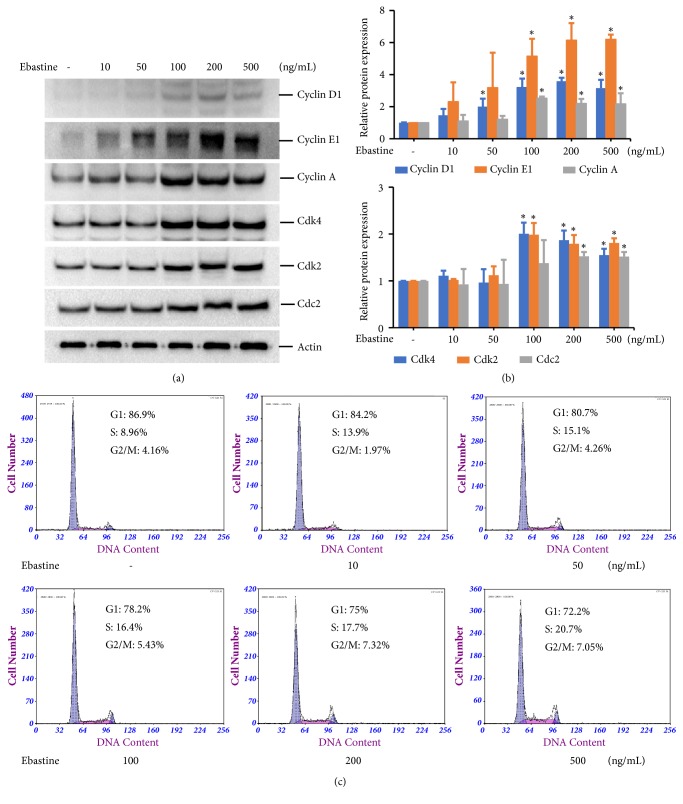
Ebastine enhanced the expression of cell cycle regulatory proteins in HFDPC. HFDPC were plated in a 10 cm dish for 24 h. The cells were serum starved for 18 h and then treated with the indicated concentrations of ebastine for 24 h. Cell lysates were prepared, and the expression levels of Cyclin D1, Cyclin E1, Cyclin A, Cdk4, Cdk2, Cdc2, and Actin were determined by immunoblotting (a). The experimental results are presented as the mean (±SD) percentage of the expression level of the indicated cell cycle regulatory proteins normalized to the expression level of the Actin protein in three independent experiments (b). Flow cytometric analysis of cell cycle parameters following 24 h of treatment with ebastine compared with untreated cells (c).

To elucidate the mechanisms underlying the regulation of cell proliferation by ebastine, we analyzed the production of cell-cycle regulatory proteins in HFDPC. We observed dose-dependent increases in Cyclin D1, Cyclin E1, and Cyclin A expression levels in ebastine-treated HFDPC (Figures 3(a) and 3(b)). Additionally, in cells treated with 100-500 ng/mL ebastine, the expression levels of Cdk4, Cdk2, and Cdc2 were increased by 1.4-2.2-fold (Figures 3(a) and 3(b)). While the Cyclin D1, Cyclin E1, Cdk4, and Cdk2 proteins are believed to be involved in the G1-to-S-phase transition, the Cyclin A and Cdc2 proteins participate in G2/M phase progression. These results suggest that ebastine induces HFDPC proliferation by enhancing the progression of the G1 and G2/M cell cycle phases. To investigate cell cycle perturbations induced by ebastine, flow cytometry analysis of propidium iodide-stained nuclei was performed. We observed dose-dependent increases in the fraction of cells in the S phase of the cell cycle for HFDPC treated with ebastine, as shown in Figure 3(c).

### 3.3. Ebastine Induced Bcl-2 Expression and Inhibited Bax Expression in HFDPC

**Figure 4:**
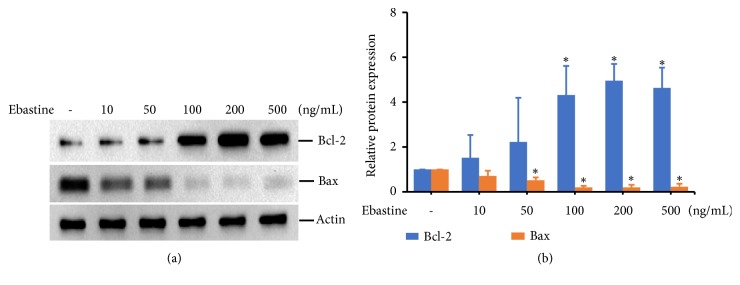
Ebastine affected apoptosis-related proteins in HFDPC. HFDPC were plated in a 10 cm dish for 24 h. The cells were serum starved for 18 h and then treated with the indicated concentrations of ebastine for 24 h. Cell lysates were prepared, and the expression levels of Bcl-2, Bax, and Actin were determined by immunoblotting (a). The experimental results are presented as the mean (±SD) percentage of the expression level of Bcl-2 and Bax proteins normalized to the expression level of the Actin protein in three independent experiments (b).

Because ebastine can induce the expression of cell-cycle regulatory proteins, we next examined whether ebastine affects the expression levels of the anti-apoptotic Bcl-2 protein and the apoptotic Bax protein. We observed a dose-dependent increase in the Bcl-2 expression level in HFDPC treated with ebastine (Figures 4(a) and 4(b)). In contrast, the levels of Bax expression were decreased when cells were treated with a high dose of ebastine (50-500 ng/mL) (Figures 4(a) and 4(b)).

### 3.4. Ebastine Induced the Phosphorylation of AKT and p44/p42 ERK in HFDPC

**Figure 5:**
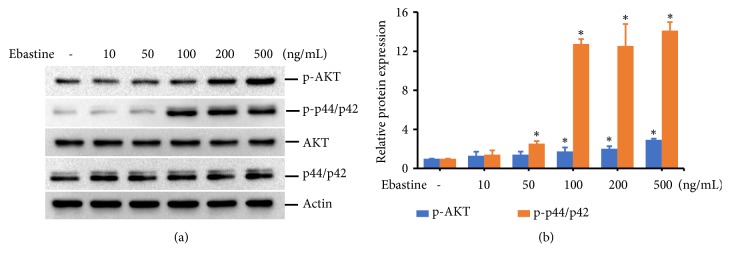
Ebastine increased p-AKT and p-p44/p42 ERK in HFDPC. HFDPC were plated in a 10-cm dish for 24 h. The cells were serum starved for 18 h and then treated with the indicated concentrations of ebastine for 24 h. Cell lysates were prepared, and the expression levels of p-AKT, total AKT, p- p44/p42 ERK, total p44/42 ERK, and Actin were determined by immunoblotting (a). The experimental results are summarized as the mean (±SD) percentage of the expression level of p-AKT and p-p44/p42 ERK proteins normalized to the expression level of Actin protein in three independent experiments (b).

Because epidermal growth factor receptor signal transduction is required for the differentiation and proliferation of papilla cells [19], we next examined the effects of ebastine on the AKT and ERK pathways in HFDPC. The protein expression level of p-AKT was slightly increased by 0.7-1.9-fold when HPDPC were treated with a high dose of ebastine (200-500 ng/mL). HPDPC treated with ebastine doses of 50-500 ng/mL showed increased expression levels of phosphorylated p44/p42 ERK by 1.4-13.1-fold (Figures 5(a) and 5(b)). No difference in the total expression levels of AKT and ERK was observed for cells treated with ebastine.

### 3.5. The ERK Inhibitor Abolished Ebastine-Induced HFDPC Proliferation

**Figure 6:**
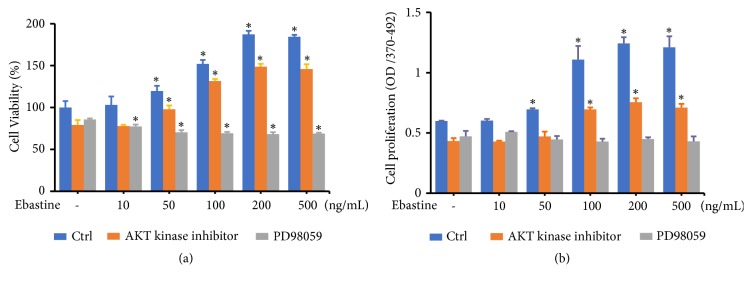
The ERK kinase inhibitor suppressed ebastine-induced cell growth of HFDPC. HFDPC were plated in triplicate in 24-well plates for 24 h. The cells were serum starved for 18 h and then treated with the indicated concentrations of ebastine in the presence or absence of the AKT1/2 kinase inhibitor (5 *μ*M) or PD98059 (50 *μ*M) for 24-48 h. Cell viability (a) and proliferation (b) were analyzed by WST-1 and bromodeoxyuridine (BrdU) assays, respectively. Representative results of three independent experiments are shown and are presented as the mean ± SD after being normalized to the control group. *∗* indicates p< 0.05.

To determine whether the AKT or ERK signaling pathways contribute to the increased HFDPC proliferation induced by ebastine, we next examined the effects of AKT and ERK kinase inhibitors on the viability of HFDPC. As shown in Figure 6(a), the number of viable cells was decreased by 20.9% and 14.4% in HFDPC treated with the AKT1/2 kinase inhibitor or PD98059 (a specific ERK kinase inhibitor), respectively. HFDPC treated with 50-500 ng/mL ebastine showed significant increases in cell viability by 19.6-77.3% and 23.6-84.1% in control cells and cells treated with the AKT1/2 kinase inhibitor, respectively (Figure 6(a)). Interestingly, in HFDPC treated with ebastine, the number of viable cells was significantly reduced by 9.7-20.3% in cells incubated with PD98059 (Figure 6(a)). Similarly, cell proliferation was decreased by 27.8% or 21.2% in HFDPC treated with the AKT1/2 kinase inhibitor or PD98059, respectively. (Figure 6(b)). In HFDPC treated with 50-500 ng/mL ebastine, cell proliferation was significantly increased by 85.2-102.2% and 60.7-74.7% in control cells and AKT1/2 kinase inhibitor-treated cells, respectively (Figure 6(b)). The observation that no doses of ebastine increased cell proliferation in HFDPC treated with PD98059 indicated that ebastine increased HFDPC proliferation via the ERK pathway.

## 4. Discussion

This study showed that ebastine at concentrations ranging from 50 to 500 ng/mL significantly increased HFDPC proliferation. The present study demonstrated that ebastine increased the expression of key cell cycle regulators, which are crucial for cell proliferation. Both AKT and ERK activity were increased in ebastine-treated HFDPC. Moreover, the ERK kinase inhibitor, but not the AKT1/2 kinase inhibitor, inhibited the proliferative effect of ebastine on HFDPC. Therefore, these results demonstrate a role for the ERK pathway in ebastine-induced HFDPC proliferation. The ebastine concentration used in this study was based on the therapeutic drug concentrations in the plasma of healthy normotensive male volunteers. The Cmax has been reported to range from 99.1±28.6 to 417±53.6 ng/mL when subjects are treated with 10 or 50 mg ebastine [18]. However, the concentrations of ebastine in the hair follicles of treated subjects have not been examined. Whether the concentrations of ebastine used in this study are similar to those of in the hair follicles of treated subjects requires further investigation.

Histamine is derived from the decarboxylation of the amino acid histidine, which occurs in endocrine cells, mast cells, and neurons [20]. Hamada et al. showed that the level of histidine decarboxylase activity increased in mice after depilation treatment and returned to the normal level within two weeks [21]. This finding suggests that histamine released from mast cells in mice may be closely related to the process of hair regrowth. It is unknown whether HFDPC can release histamine. Regardless of the quantity of histamine secreted by HFDPC, the amount of histamine in HFDPC basal medium was low. Further investigation is needed to determine whether ebastine itself or its metabolites [22], including desalkylebastine, hydroxyebastine, and carebastine, are involved in the AKT/ERK pathway. Although both ebastine and carebastine possess antihistamine H1 activity [23], determining whether other receptors activated by ebastine or its metabolites are involved in the AKT and ERK pathways merits further investigation.

Ebastine was developed to block the histamine H1 receptor in the treatment of allergic inflammatory reactions, but many histamine receptors in cells may be targets of antihistamines. Histamine can stimulate human carcinoma cell proliferation and chemotactic ability through the histamine H1 receptor [24]. The histamine H1 receptor has also been shown to suppress epidermal keratinocyte differentiation, and the histamine H1 receptor agonist 2-pyridylethylamine has the same effects as histamine [25]. Furthermore, the histamine H1 receptor antagonist, but not antagonists of the other three histamine receptors, has been shown to suppress the effects of histamine on loricrin and filaggrin expression in differentiating monolayer cultures. These previous findings together with our studies indicate that the histamine H1 receptor not only plays a significant role in allergic inflammation but also mediates cell differentiation and cell growth.

A limited number of studies reported the literature regarding the relationship between histamine receptor blockade and growth factor signaling pathways. In contrast, some research results have discussed the role that histamine receptors play in cell growth. Cholangiocarcinoma cells proliferate through the histamine-induced upregulation of cAMP and calcium/PKC, which leads to increased ERK activity and cancer cell growth [26]. Additionally, spinal ERK activation has also been observed in histamine-induced acute itching [27]. In contrast, histamine inhibits the proliferation of human prostatic adenocarcinoma DU-145 cells through the activation of H1 receptors [28]. Histamine H4 receptor has also been shown to inhibit the proliferation of Leydig tumor cells [29]. These results suggest that the content of various histamine-bound receptors or receptor binding proteins causes the differences between histamine-induced signal transduction in different cell types.

Alopecia areata is considered a cell-mediated autoimmune disease that causes hair loss on the scalp or the body [30, 31]. The most characteristic histopathological change in alopecia areata is hair bulbs that are surrounded by bulbar lymphocytes, which are composed of both CD4^+^ and CD8^+^ T cells [32]. Collapse of immune privilege in hair follicles may induce Th1 cytokine or chemokine expression, including IFN-*γ* or CXCL10, in hair bulbs. This is speculated to contribute to the development of aopecia areata [33–35]. In addition to the inhibitory effect on Th2-type cytokine production, ebastine can also inhibit T cell migration [16]. Decreased T cell infiltration has been observed in ebastine-treated mice, suggesting that ebastine can combat alopecia areata, and has been implicated in immune regulation [14].

Over the years, antihistamine drugs have been shown to completely reverse baldness [36, 37]. Some studies attempting to explain the possible effects of antihistamines on hair regrowth have attributed hair regrowth to the immunoregulatory effect [14, 15]. In addition to immune regulation by ebastine to treat alopecia areata, we observed that ebastine regulated HFDPC proliferation via activation of the ERK pathway might be related to hair regrowth. Further investigation of ebastine-induced hair growth* in vivo* should be conducted, which may offer new targets for treatments to reverse baldness.

## Data Availability

All data used to support the findings of this study are included within the article.
